# Comparison of characteristics and injury patterns between minor and adult patients seeking emergency cosmetic suture for facial lacerations: data from a tertiary plastic surgery hospital in Beijing, China

**DOI:** 10.3389/fpubh.2025.1656790

**Published:** 2025-12-02

**Authors:** Ziyuan Chen, Xiaoyue Mao, Huaizheng Wu, Yakun Shi, Chao Nian

**Affiliations:** 1 Department of Plastic Surgery, Plastic Surgery Hospital, CAMS & PUMC, Beijing, China; 2 Outpatient and Emergency Operating Room, Plastic Surgery Hospital, CAMS & PUMC, Beijing, China; 3 Information Technology Department, Plastic Surgery Hospital, CAMS & PUMC, Beijing, China; 4 Central Operating Room, Plastic Surgery Hospital, CAMS & PUMC, Beijing, China

**Keywords:** emergency, cosmetic suture, facial lacerations, characteristics, injury patterns, minors, adults

## Abstract

**Background:**

Emergency cosmetic suture is frequently required by patients with soft tissue injuries. Understanding the epidemiology and injury features of different age groups in this population facilitates wound management and prevention. Based on large patient volume from a tertiary plastic surgery hospital in Beijing, our study aimed to compare the characteristics and injury patterns between minor and adult patients seeking emergency cosmetic suture for facial lacerations.

**Methods:**

This is a retrospective study on patients undergoing emergency cosmetic suture in Plastic Surgery Hospital, CAMS & PUMC from August 2022 to July 2024. Data including demographics, time metrics, wound number and size, cause and position of injury, injection of tetanus immunoglobulin were collected.

**Results:**

Totally 18,412 patients were included, comprising 12,613 (68.5%) minors and 5,799 (31.5%) adults. Median age was 7 (IQR 4–25) years. Male percentages were 61.7% in minors vs. 49.5% in adults. Clinical workload was heavier from March to October, on weekends and during 20:00–23:00 of a day. Injury to door time, consultation to operation time and operation time were longer in adults. Adults also tended to have multiple, longer lacerations and lacerations related to traffic or assault. Common positions of injury were frontal region (33.1%), mental region (22.2%), periorbital region (21.1%) in minors and periorbital region (25.6%), frontal region (24.4%), perioral region (16.3%) in adults. Injection of tetanus immunoglobulin was more frequent in adults.

**Conclusion:**

Characteristics and injury patterns of minor and adult patients were different in many aspects. The findings may contribute to preventing injuries among targeted age groups, assessing wound condition and optimizing working shift allocation.

## 1 Introduction

Soft tissue injuries are frequently managed in the emergency department (ED), accounting for 7–10% of total visits (1, 2). Common types of soft tissue injuries include contusions, abrasions, bruises, hematomas, lacerations, incisions and burns (3, 4). Lacerations make up 41.3–56.5% of all cases (5, 6), and usually require surgical suture to repair. Etiology of soft tissue trauma includes accidents and falls, sports, motor vehicle accidents, assaults and violence, and occupational causes (7, 8). Many previous studies each focused on a specific age group and reported diverse patterns of injury (1, 9–11), while studies directly comparing features of minor and adult patients in a large population were scarce.

Injuries to the face hold an important position due to their esthetic and functional implications (12). Since formation of cicatrices may impair individual’s appearance and cause subsequent psychological distress (13, 14), the cosmetic outcome of wound repair especially in the facial region is considered particularly critical for injured patients or their guardians (15–18). Plastic surgery consultations are frequently required, especially in pediatric ED (19, 20). Patients seeking cosmetic repair of facial wounds form a new cohort. Investigating the epidemiological characteristics of them is meaningful and necessary, which may facilitate diagnosis, treatment and prevention of injuries. However, limited availability of on-call plastic surgeons and ambiguous consultative obligation (21) restricted the volume of patients undergoing cosmetic suture, leading to scarcity of reports on this cohort.

Plastic Surgery Hospital, Chinese Academy of Medical Sciences & Peking Union Medical College (CAMS & PUMC) is China’s earliest tertiary hospital specialized in plastic and reconstructive surgery, with over 300,000 outpatient visits annually. Twenty-four-hour emergency cosmetic suture is routinely provided by a team of on-call plastic surgeons, accomplishing over 10,000 cases of emergency wound repair per year. Based on abundant data, our study aimed to compare the characteristics and injury patterns between minor and adult patients seeking emergency cosmetic suture for facial lacerations.

## 2 Methods

This is a retrospective observational study based on data of patients who visited ED in Plastic Surgery Hospital, CAMS & PUMC in two consecutive years (from August 2022 to July 2024). Eligible patients were those who presented with facial lacerations suitable for primary suture and without other complications that required urgent management. Collected patient data included demographics (gender, age); time points (time of injury, time of registry, time of diagnosis, beginning and finishing time of operation), wound information (cause, position, number and length), and injection of tetanus immunoglobulin. Gender, age, time of registry and time of diagnosis were identified in the hospital information system (HIS). Time of injury, cause, position number and length of wounds were extracted from history of present illness. In cases with multiple wounds, position and length of the main injury were documented. Beginning and finishing time of operation were extracted from operative records. Injection of tetanus immunoglobulin was identified from history of present illness and the medical order sheets. Patients missing the above information were excluded. Two researchers worked together during data collection, with one researcher in charge of data entry and the other performing double-check to ensure quality control.

**Figure 1:**
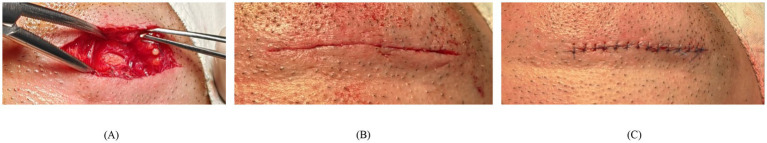
**(A)** A typical laceration in the mental region. The wound was 4.5 cm long, involving skin, muscle and periosteum layers. **(B)** After performing the central step—tension-reducing buried subcuticular suture using absorbable sutures, the wound was already closed neatly. **(C)** Cosmetic suture was finished with careful epidermal approximation using non-absorbable monofilament sutures.

In this study, cosmetic suture was defined as any primary closure of facial lacerations performed in operation rooms by on-call plastic surgeons with at least 2 years of clinical experience (totally about 40 attendings/senior residents during the 2 years). A typical facial laceration was shown in Figure 1. Standard procedures of cosmetic suture included:

1. After preliminary assessment, peri-wound skin was disinfected by iodophor. Wounds were irrigated with sterile saline. Field of operation was draped with fenestrated sheet.
2. Local infiltration or nerve block anesthesia was conducted using lidocaine and 1:400000 epinephrine.
3. After thorough debridement, wound edges were finely trimmed. Subcutaneous dissection was performed to reduce wound tension when necessary.
4. Soft tissue injury was repaired layer by layer. Tension-reducing buried subcuticular suture was routinely performed using absorbable sutures (4–0, 5–0 or 6–0), followed by meticulous epidermal approximation with non-absorbable monofilament sutures (6–0 or 7–0).
5. Absorbent dressings or non-adherent dressings were selected according to the condition of wounds. Topical antiseptics were recommended. Scar prevention with silicone gel was recommended after suture removal.

Statistical analyses were performed using Stata software (version 18.0). Pearson’s chi-squared test and Wilcoxon rank-sum test were used to test differences of discrete variables between groups. Kruskal-Wallis rank-sum test was used to test differences of continuous variables between groups. The study received ethics approval from the Medical Ethics Review Board of Plastic Surgery Hospital, CAMS & PUMC.

## 3 Results

### 3.1 Demographics

In the study period, 18,412 patients who underwent cosmetic suture for facial lacerations were included. Age of the population ranged from 0 to 94 years, with a median of 7 (IQR 4–25) years. The majority of injured patients were male, accounting for 57.9% (*n* = 10,655).

### 3.2 Distribution of age and gender

**Figure 2:**
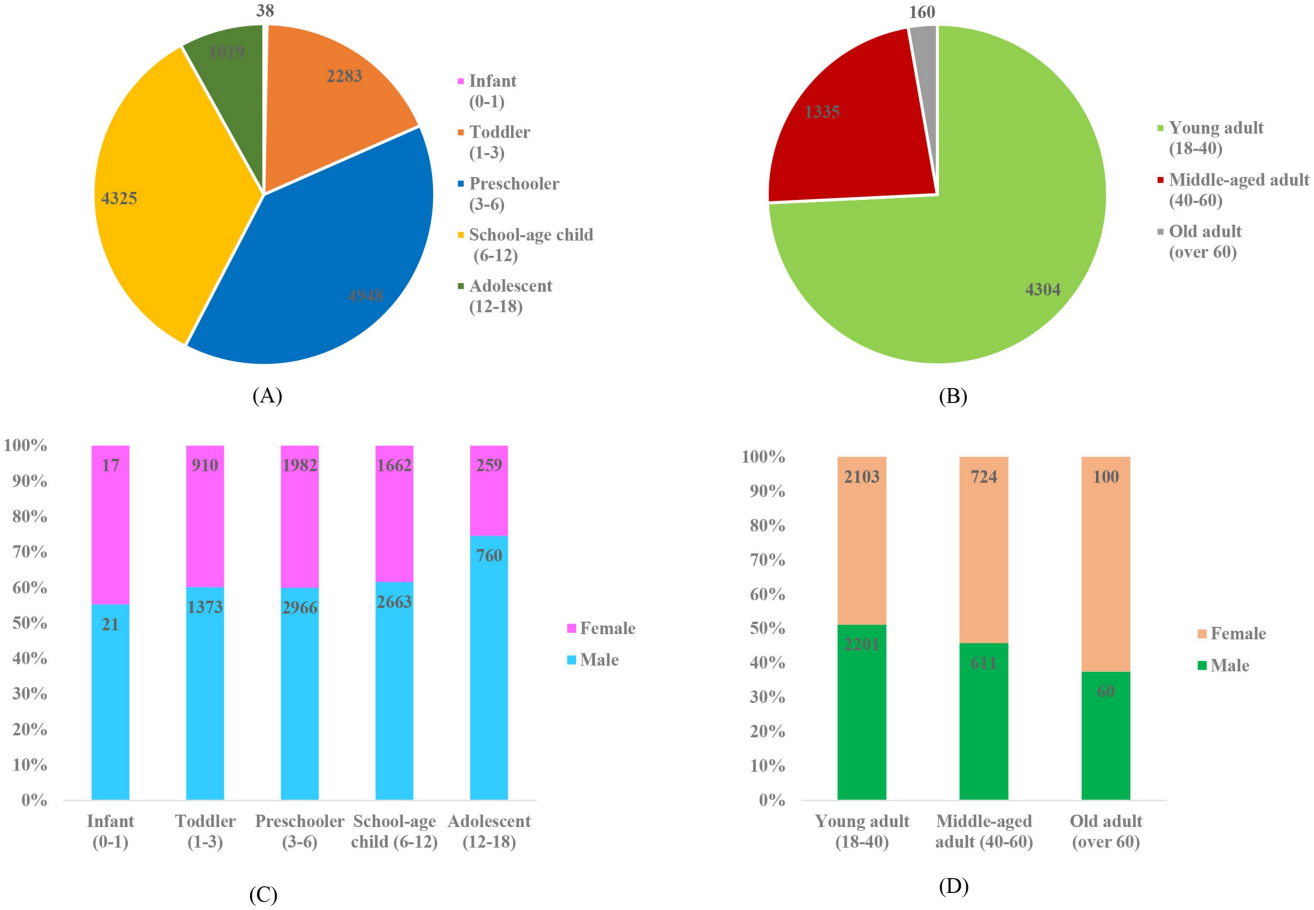
**(A)** Age composition of minor patients. **(B)** Age composition of adult patients. **(C)** Gender composition of minor patients. **(D)** Gender composition of adult patients.

Of the 18,412 patients, 12,613 (68.5%) were minors (age <18 years) and 5,799 (31.5%) were adults (age≥18 years). Median age of minors and adults were 5 (IQR 3–7) years and 33 (IQR 26–40) years, respectively. Proportion of male gender in minor patients was significantly higher than adult patients (61.7% vs. 49.5%, *p* < 0.001). Minor and adult patients’ age was divided into subgroups for further analysis. Distribution of age and gender in each subgroup was illustrated in Figure 2. Preschoolers and school-age children made up largest proportions of minors. Young adults were dominant in adult group.

In minor patients, percentage of male gender gradually rose from 55% (infant subgroup) to over 60% (toddler, preschooler, school-age child subgroup) and reached 75% in adolescent subgroup. On the contrary, the adult patients’ male percentage dropped from 51% (young adult subgroup) to 46% (middle-aged adult subgroup) and finally 38% in old adult subgroup.

### 3.3 Comparison of visiting time and other time metrics

**Figure 3:**
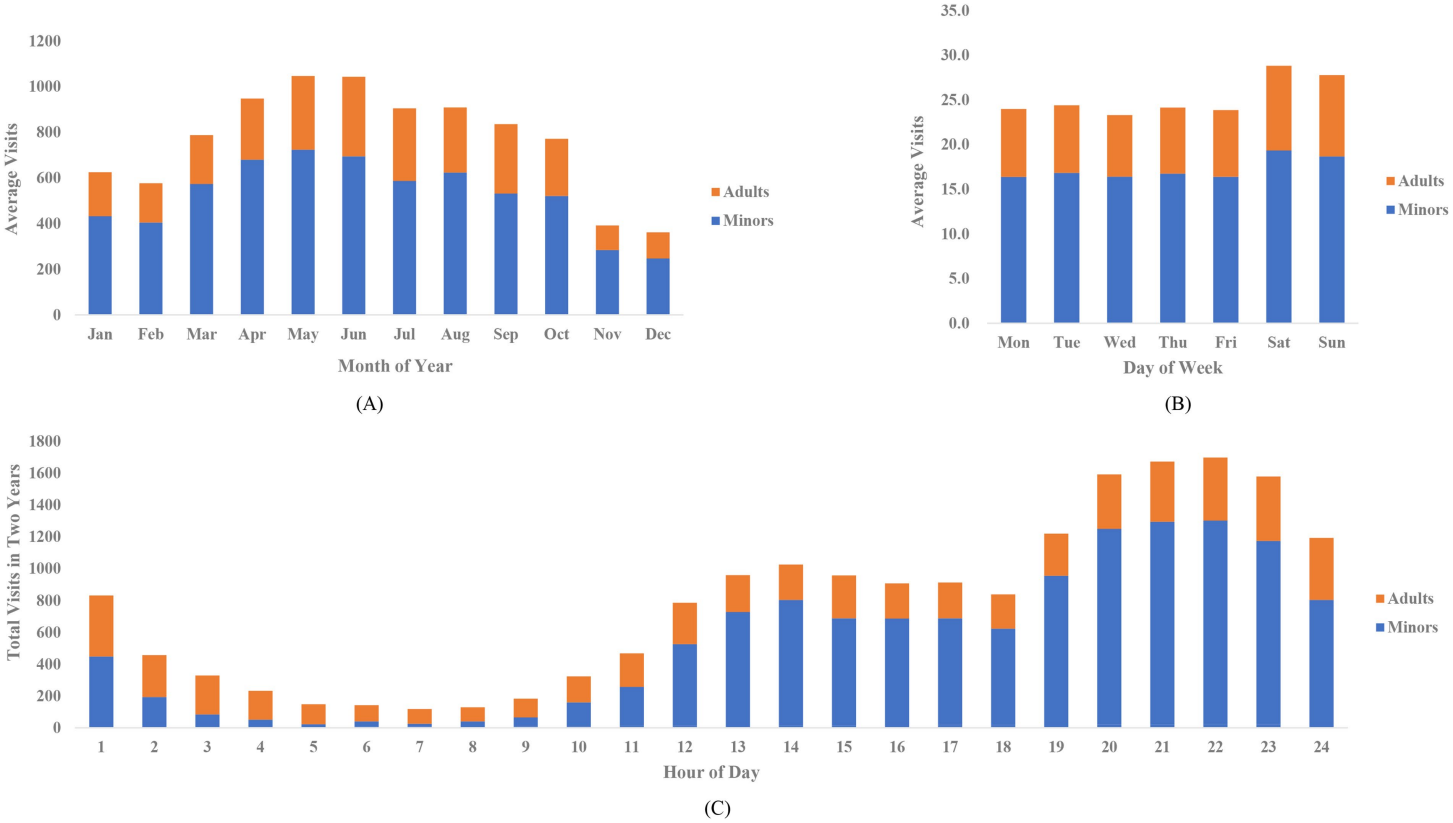
**(A)** Average patient visits in each month of a year. **(B)** Average patient visits on each day of a week. **(C)** Total patient visits in each hour of a day.

Time of visit was determined by time of registry in the HIS system. Distributions of patient visits in each month of a year, each day of a week, and each hour of a day were depicted in Figure 3. From August 2022 to July 2024, a monthly average of 767 patients received cosmetic suture of facial lacerations in Plastic Surgery Hospital, CAMS & PUMC. May and June had the most average visits (over 1,000) while November and December had the least (less than 400). In average, 25–26 patients were operated each day. Weekday and weekend averages were 24 patients and 28 patients, respectively. Patient visits occurred in each hour of a day revealed busy hours (20:00–23:00) and relatively free (2:00–11:00) hours.

Distribution of minors’ and adults’ visits remained similar from January to December and from Monday to Sunday. However, within a day, proportion of adult visits significantly increased during 1:00–11:00. Adult patients outnumbered minor patients during 2:00–10:00.

Other time parameters were also shown in Table 1. Minor patients and adult patients had statistically significant difference in injury to door time (*p* < 0.001), consultation to operation time (p < 0.001) and operation time (p < 0.001). It took more time for adults to visit our hospital after being injured. Their waiting time between consultation and operation was longer. Duration of suture was also longer.

**Table 1:** Demographics and time metrics of patients with facial lacerations.

	Minors (*n* = 12,613)	Adults (*n* = 5,799)	Total (*n* = 18,412)	*p* value
Age, years (median, IQR)	5 (3–7)	33 (26–40)	7 (4–25)	
Gender, male (%)	7,783 (61.7)	2,872 (49.5)	10,655 (57.9)	<0.001
Injury to door, hours (median, IQR)	2 (2–3)	3 (2–5)	2 (2–4)	<0.001
Door to consultation, minutes (median, IQR)	10 (4–22)	10 (4–23)	10 (4–23)	0.06
Consultation to operation, minutes (median, IQR)	24 (16–40)	25 (17–41)	24 (17–41)	<0.001
Operation time, minutes (mean±SD)	16.0 ± 5.6	19.0 ± 11.1	17.0 ± 7.9	<0.001

### 3.4 Comparison of wound number and size

**Figure 4:**
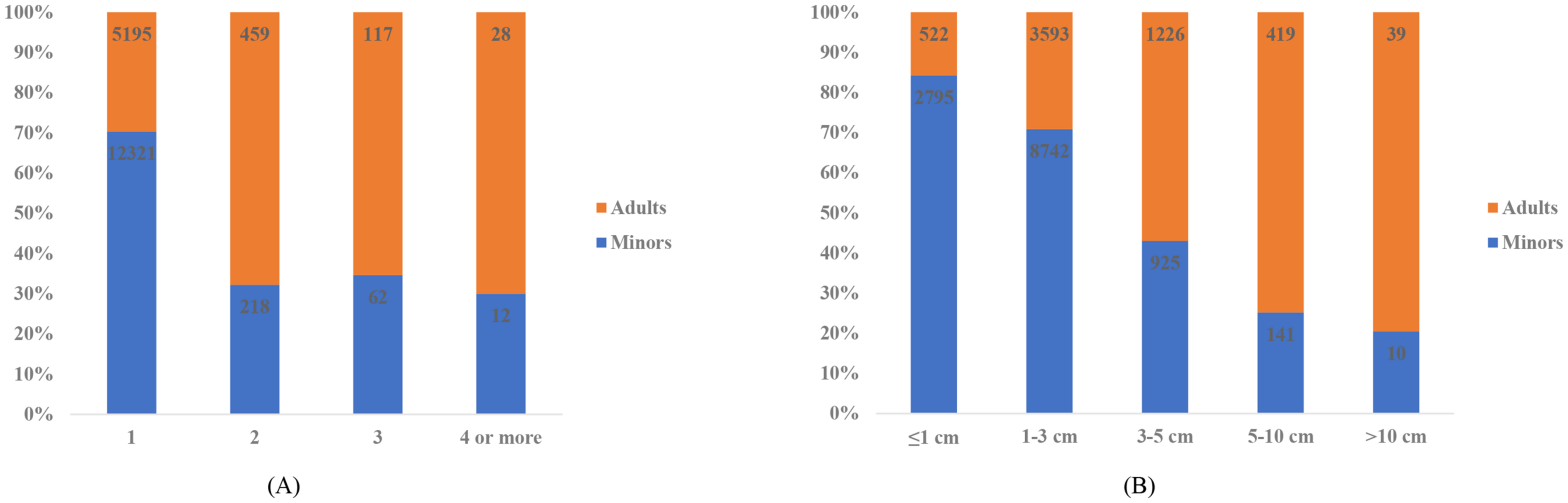
**(A)** Minor/adult distribution of wound number. **(B)** Minor/adult distribution of wound size.

Number and length of wounds were categorized in Table 2. Although single wound and wound length of 1-3 cm were dominant in both minor and adult patients, significant differences existed between the two groups (*p* < 0.001). Minor/adult ratios of each category of wound number and size were shown in Figure 4. Adults accounted for approximately 70% of patients with multiple wounds. Besides, the proportion of adults increased with wound length.

**Table 2:** Number, length, and causes of wounds.

	Minors (*n* = 12,613)	Adults (*n* = 5,799)	Total (*n* = 18,412)	*p* value
**Number of wounds**				<0.001
1	12,321 (97.7%)	5,195 (89.6%)	17,516 (95.1%)	
2	218 (1.7%)	459 (7.9%)	677 (3.7%)	
3	62 (0.5%)	117 (2.0%)	179 (1.0%)	
≥4	12 (0.1%)	28 (0.5%)	40 (0.2%)	
**Wound length**				<0.001
≤1 cm	2,795 (22.2%)	522 (9.0%)	3,317 (18.0%)	
1–3 cm	8,742 (69.3%)	3,593 (62.0%)	12,335 (67.0%)	
3–5 cm	925 (7.3%)	1,226 (21.1%)	2,151 (11.7%)	
5–10 cm	141 (1.1%)	419 (7.2%)	560 (3.0%)	
>10 cm	10 (<0.1%)	39 (0.7%)	49 (0.3%)	
**Causes of injury**				<0.001
Fall/Accident	12,394 (98.3%)	4,866 (83.9%)	17,260 (93.7%)	
Assault	88 (0.7%)	365 (6.3%)	453 (2.5%)	
Traffic	109 (0.9%)	491 (8.5%)	600 (3.3%)	
Sport	8 (<0.1%)	16 (0.3%)	24 (0.1%)	
Animal	10 (<0.1%)	31 (0.5%)	41 (0.2%)	
Work	0 (0%)	27 (0.5%)	27 (0.2%)	
Other	4 (<0.1%)	3 (<0.1%)	7 (<0.1%)	

### 3.5 Comparison of injury causes and positions

As shown in Table 2, the leading cause of both minor and patients was fall/accident. The other two common causes were traffic and assault. Sport, animal and work-related injuries were also documented. Of note, numbers of adults injured by assault and traffic were more than 4 times higher than that of minors.

Detailed positions of facial lacerations were summarized in Table 3. In whole population, three most common positions of injury were frontal region (30.3%), periorbital region (22.5%) and mental region (19.9%). In minor patients, three most common locations were frontal region (33.1%), mental region (22.2%) and periorbital region (21.1%) in comparison with periorbital region (25.6%), frontal region (24.4%) and perioral region (16.3%) in adult patients. We subdivided periorbital region and perioral region into more accurate anatomical units and found that lacerations to periorbital region most frequently occurred on eyebrow and upper eyelid in both age groups. Incidence of upper lip injury was evidently higher than that of lower lip in the adult group only. Documented numbers of lacerations to scalp, temporal region, zygomatic region and auricle were relatively small.

**Table 3:** Detailed positions of facial lacerations.

	Minors (*n* = 12,613)	Adults (*n* = 5,799)	Total (*n* = 18,412)	*p* value
**Positions of injury**				<0.001
Scalp	274 (2.2%)	72 (1.2%)	346 (1.9%)	
Frontal region	4,175 (33.1%)	1,412 (24.4%)	5,587 (30.3%)	
Periorbital region	2,665 (21.1%)	1,485 (25.6%)	4,150 (22.5%)	
Eyebrow	1,324 (10.5%)	745 (12.8%)	2069 (11.2%)	
Upper eyelid	1,008 (8.0%)	538 (9.3%)	1,546 (8.4%)	
Lower eyelid	111 (0.9%)	117 (2.0%)	228 (1.2%)	
Medial canthus	17 (0.1%)	15 (0.3%)	32 (0.2%)	
Lateral canthus	205 (1.6%)	70 (1.2%)	275 (1.5%)	
Temporal region	154 (1.2%)	106 (1.8%)	260 (1.4%)	
Nasal region	593 (4.7%)	344 (5.9%)	937 (5.1%)	
Zygomatic region	80 (0.6%)	83 (1.4%)	163 (0.9%)	
Cheek	497 (3.9%)	399 (6.9%)	896 (4.9%)	
Perioral region	1,154 (9.2%)	947 (16.3%)	2,101 (11.4%)	
Upper lip	541 (4.3%)	721 (12.4%)	1,262 (6.9%)	
Lower lip	588 (4.7%)	209 (3.6%)	797 (4.3%)	
Not specified	25 (0.2%)	17 (0.3%)	42 (0.2%)	
Mental region	2,797 (22.2%)	869 (15.0%)	3,666 (19.9%)	
Auricle	224 (1.8%)	82 (1.4%)	306 (1.7%)	

### 3.6 Comparison of tetanus immunoglobulin injection

Tetanus immunoglobulin injection was given to 38.6% of all patients. Three-fourth of them were injected after arriving to our hospital, no matter minors or adults. However, many preschoolers were exempt from injection due to recent DPT vaccination, leading to significantly lower injection rate in minor patients (*p* < 0.001) (Table 4).

**Table 4:** Injection of tetanus immunoglobulin.

	Minors (*n* = 12,613)	Adults (*n* = 5,799)	Total (*n* = 18,412)	*p* value
Unnecessary	8,551 (67.8%)	2,762 (47.6%)	11,313 (61.4%)	<0.001
Given by our center	3,190 (25.3%)	2,401 (41.4%)	5,591 (30.4%)	
Given earlier by other hospitals	872 (6.9%)	636 (11.0%)	1,508 (8.2%)	

## 4 Discussion

By analyzing 18,412 patients who visited our ED from August 2022 to July 2024, this study comprehensively summarized characteristics of the cohort seeking emergency cosmetic suture for facial lacerations. Injury patterns of minor and adult patients were compared in many aspects. Although emergency cosmetic suture is provided for all ages in our center, over two-thirds of the population were minor patients. The proportions of preschoolers and school age children were particularly high, partly because of the special attention guardians pay for facial lacerations to their children (22, 23).

Another socioeconomic factor relevant to the age distribution is commercial accident insurance, since cosmetic suture is not covered by basic medical insurance. According to complexity of repair, cosmetic suture for a 1–3 cm facial laceration may cost up to 6,000 RMB (approximately 850 USD). Economically disadvantaged self-paying patients may be inclined to receive normal suture in general surgery ED. In China, many insurance companies provide ‘student safety insurance’ with affordable premium (100–200 RMB per year) and insurance coverage of 20,000–100,000 RMB. Although not compulsory, the Ministry of Education encourages primary school and middle school students to participate such accident insurance with public welfare characteristics (24). The national wide insurance rate was not revealed, yet certain region reported insurance rate of over 95% (25–27). ‘Student safety insurance’ contributed to higher proportion of minors seeking cosmetic suture.

Studies reported male proportion of 64.4–66% in patients with soft tissue injuries who visited regular ED (22, 28–30). In our study, male percentage of minor patients (62%) was comparable to previous studies. Remarkably, adolescent subgroup had the highest male proportion of 74.5%. Preventive measures should be targeted on this high-risk group. Schools must actively conduct safety education and take effective precautions. In contrast, only 49.5% of adult patients were male. With the increase of age, female gender became dominant. Researchers have pointed out that females had much stronger motivations and pursuit for aesthetic surgeries (31, 32). Our study confirmed their stronger willingness to seek emergency cosmetic suture for injury as well.

Distributions of patient visits were related to injury patterns. Increased frequency of outdoor activities was a critical reason for larger patient volume during summertime and weekends. Within a day, number of visits reached its first peak at around 14:00 and a higher second peak at around 22:00. We considered the first peak as a result of accidents happened to minor patients at school, which was supported by a study on circadian rhythms of pediatric trauma (33). The second peak was usually associated with increased traffic accidents and assaults of adult patients.

During 2:00–10:00, the majority were adult patients, many of whom consumed alcohol before injured. Alcohol is a risk factor of both intentional injuries (assaults) and unintentional injuries (falls, traffic accidents and workplace injuries) (34–36), increasing incidence of adult injury at night. From our experience, drunk patients often spent longer time getting to the hospital and needed extra rest before they could cooperate during surgery, extending door to consultation and consultation to operation time in adult group.

Distribution of patient visits reflected burden of clinical work and provided instructions on optimizing arrangement of medical facilities. In our practice, a swing shift (12:00–24:00) was arranged in addition to day shift (8:00–18:00) and night shift (18:00–8:00 next day) to relieve workload during March to October. This arrangement effectively shortened patients’ waiting duration, enabling a 10-min median door to consultation time and a 24-min median consultation to operation time, elevating patient satisfaction.

Multiple wounds and longer lacerations were more frequently seen in adult patients. Number and size to a large extent reflected severity and complexity of injury and hence difficulty in suture. In this sense, longer operation duration also suggested more severe and complex wounds in the adult group.

The leading cause of injury was fall/accident in both minors and adults, in accordance with many previous studies (1, 5, 9). Nonetheless, proportion of fall/accident in our study population reached a much higher level of 90% since other common causes such as assaults and road accidents were more likely to cause severe trauma, in which case priority was taking first aid measures rather than seeking cosmetic suture.

Frontal region, mental region and periorbital region were three most common positions of lacerations in minors, adding up to 76.4%. Periorbital region, frontal region and perioral region were most susceptible in adults, adding up to 66.3%. Such patterns of injury call for particular protection to these facial positions in certain age groups.

Our research had limitations. This was a single-center retrospective study with data from a tertiary plastic surgery hospital in Beijing. The results may not extend to medical institutions in rural areas. Data were obtained from medical records written by different on-call surgeons who may have different manners of describing injuries. Large sample size and proper classification of data items such as length range of lacerations in our study mitigated potential bias. Causes of injury were not specifically classified, hindering deeper analysis of etiology. Socioeconomic conditions of injured patients were not stratified for comparison. Outcomes of suture were not evaluated. Therefore, this study merely focused on epidemiological characteristics and injury patterns rather than postoperative recovery. Future studies may investigate complications and patient satisfaction of cosmetic suture in minor and adult patients.

## 5 Conclusion

Characteristics and injury patterns of minor and adult patients seeking cosmetic suture for facial lacerations were different. Minors constituted two-thirds of total population. Adult females were more motivated to seek cosmetic suture. Distribution of visiting time revealed underlying mechanisms. Adult patients were associated with more severe injuries. Common causes and positions of injuries in each age group provided guidance on prevention, diagnosis and treatment.

## Data Availability

The raw data supporting the conclusions of this article are available from the corresponding author by request.
